# Lateral Electron and Hole Hopping between Dyes on Mesoporous ZrO_2_: Unexpected Influence of Solvents with a Low Dielectric Constant

**DOI:** 10.1021/jacs.3c01333

**Published:** 2023-05-01

**Authors:** Sina Wrede, Bin Cai, Amol Kumar, Sascha Ott, Haining Tian

**Affiliations:** † Department of Chemistry-Ångström Laboratory, Uppsala University, SE-75120 Uppsala, Sweden

## Abstract

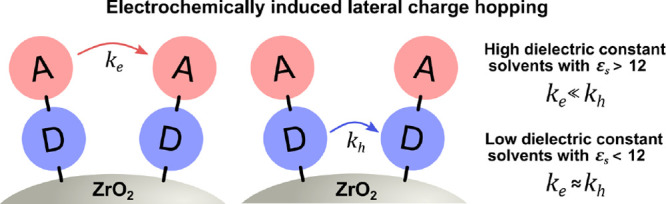

Lateral intermolecular charge transfer between photosensitizers on metal oxide substrates is important for the understanding on the overall working principles of dye-sensitized systems. Such studies usually concentrate on either hole or electron transfer separately and are conducted in solvents with a high dielectric constant (ε_*s*_) that are known, however, to show a drastic decrease of the local dielectric constant close to the metal oxide surface. In the present study, both hole and electron hopping between organic donor–acceptor photosensitizers was experimentally investigated on PB6 dye-sensitized mesoporous ZrO_2_ films. The donor (close to the surface) and acceptor (away from surface) subunit of the PB6 dye were observed to be involved in hole and electron hopping, respectively. Hole and electron transfer kinetics were found to differ remarkably in high-ε_*s*_ solvents, but similar in solvents with ε_*s*_ < 12. This finding indicates that low-ε_*s*_ solvents maintain similar local dielectric constant values close to, and further away from, the semiconductor surface, which is different from the previously observed behavior of high dielectric constant solvents at a metal oxide interface.

Dye-sensitized mesoporous metal oxides have been widely used in solar energy conversion and storage devices such as dye-sensitized solar cells and dye-sensitized solar fuel devices.^1,2^ Intermolecular self-exchange electron transfer reactions between immobilized dyes on the mesoporous metal oxide support, also called charge “hopping”, have been studied to understand the fundamental charge transfer pathways that can occur during energy conversion.^3^ Charge hopping between dyes such as metal complexes (for example Ru,^4−8^ Zn,^6^ and Os^9^ complexes), fullerene derivatives,^10^ and organic photosensitizers^6,11^ on different metal oxide supports has been investigated. A typical mesoporous support for charge hopping studies is the n-type semiconductor TiO_2_,^4−9^ but insulating mesoporous oxides such as ZrO_2_ and Al_2_O_3_^10^ and the p-type semiconductor NiO^11,12^ have also been utilized. However, hole hopping is typically studied on TiO_2_ and electron hopping on NiO, which makes direct comparison between hole and electron hopping rates challenging due to different dye loading and mesoporous film properties. Even though Shan et al.^12^ have studied hole and electron hopping of a dye–catalyst assembly on different semiconductor supports, lateral hole and electron hopping in a single photosensitizer/metal oxide system has not been investigated in the literature. In addition to possible intrinsic differences in electron and hole hopping rates, such a system would also make it possible to investigate how hopping rates may also be affected by other factors such as solvent dielectric constant that is known to differ depending on distance to the semiconductor surface.

Herein, we report an organic donor–acceptor dye (PB6) with a triphenylamine (TPA) donor and perylene monoimide (PMI) acceptor moiety on mesoporous ZrO_2_, which is used to study both electron and hole hopping between dyes across the surface (Figure 1). The wide bandgap semiconductor ZrO_2_ makes it possible to monitor both electrochemically induced hole and electron hopping on the same support since the dye potentials lie within the bandgap. The excellent reversibility and stability of PB6 during reduction and oxidation allows the investigation of the electron and hole hopping kinetics as a function of solvent bulk dielectric constants (ε_*s*_). In the literature, acetonitrile is typically used as the electrolyte solvent, though some studies in water and other solvents exist, all with an ε_*s*_ > 25.^13−16^ Such solvents are known to exhibit a drastic decrease in dielectric constant close to the metal oxide support, which results in faster charge hopping kinetics close to the surface due to a near-zero outer-sphere solvent reorganization energy.^13−15^ We observe that, in contrast to solvents with a higher ε_*s*_, solvents with a lower ε_*s*_, such as dichloromethane and tetrahydrofuran, give rise to similar charge transfer kinetics both further away from and close to the surface. This strongly suggests that the observed electron and hole transfer kinetics are not intrinsically different but instead a result of the position of the subunits of the dye that participate in the hopping process and the local environment they experience.

**Figure 1:**
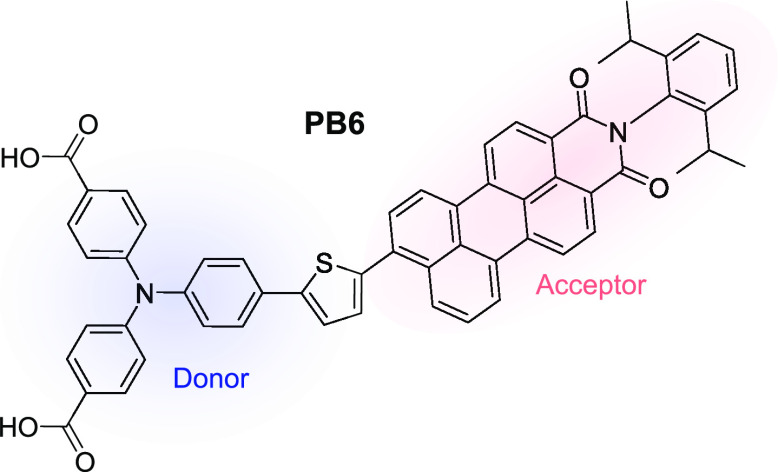
Molecular structure of the PB6 photosensitizer.

Electrochemically induced hopping, a well-established method to study charge hopping across a surface,^6,9,17−20^ was chosen for this study since photoinduced charge hopping is unsuitable due to a lack of charge injection into ZrO_2_. In electrochemically induced hopping on an insulator or semiconductor, the support itself does not transport the charge to the immobilized molecules. Instead, the hopping process is initiated only by the small fraction of molecules close to, or in contact with, the conductive fluorine-doped tin oxide (FTO) glass substrate (Figure 2). The charge transport initiated by the potential step can be monitored by absorption changes in the UV–vis spectrum of the film. From the kinetic traces, the apparent diffusion coefficient (*D*_*app*_) of charge hopping through the mesoporous film can be determined from analysis with a modified version of the Cottrell equation (for a detailed explanation, see SI).^20−22^

**Figure 2:**
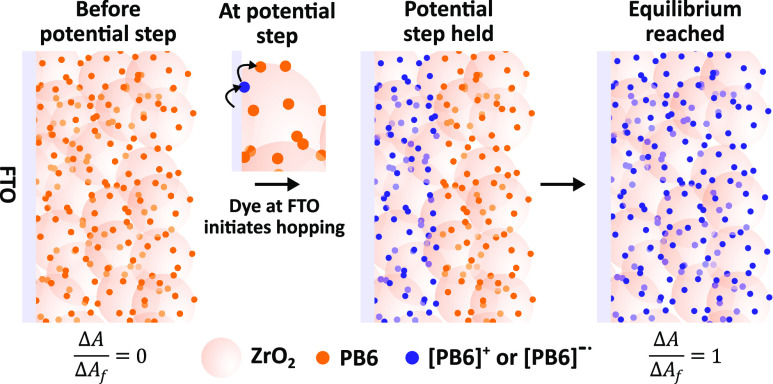
Schematic illustration of the sensitized ZrO_2_ film during chronoabsorptometry experiment.

The prepared dye-sensitized ZrO_2_ films (ZrO_2_-PB6) were used as working electrode in the optical path in a three-electrode cell with a Ag/AgNO_3_ reference electrode and a platinum wire counter electrode. The chosen potential steps for electrochemically induced hole or electron hopping were +0.87 V and −1.37 V vs Ag/AgNO_3_, respectively (full experimental details in SI).

Upon the application of the respective potential steps for electrochemically induced hole or electron hopping, a change in absorption with distinct features could be observed (Figure 3A,B). In its ground state, the PB6 dye has two major absorption features, at approximately 355 and 526 nm (see SI, Figure S1). Most noticeably, upon reduction or oxidation of the dye, one or the other feature displays a decrease in absorbance, depending on the involved subunit which is the major contributor of the spectral feature; the PMI acceptor has a larger contribution to the 526 nm feature, which is typical for PMI,^23^ whereas the TPA donor contributes more to the 355 nm feature, which is similar to the absorption feature of TPA analogues in the literature.^24^ If a positive potential step is applied, an absorption decrease at 355 nm can be observed that is absent when a negative potential step is applied. This absorption decrease indicates that the TPA donor unit becomes oxidized and that the charge is localized. A broad absorption feature between 650 and 1000 nm appears that can be assigned to oxidized PB6 (PB6^+^). Upon the application of a negative potential step, a decrease of the 525 nm absorption feature and the typical rise of the three absorption features of the PMI subunit^11^ can be seen for reduced PB6 (PB6^•–^), at 634, 744, and 842 nm (Figure 3B). The spectroscopic features of PB6^+^ and PB6^•–^ support the presumption that hole and electron hopping between PB6 immobilized on the ZrO_2_ surface occurs primarily between the respective donor and acceptor subunits.

**Figure 3:**
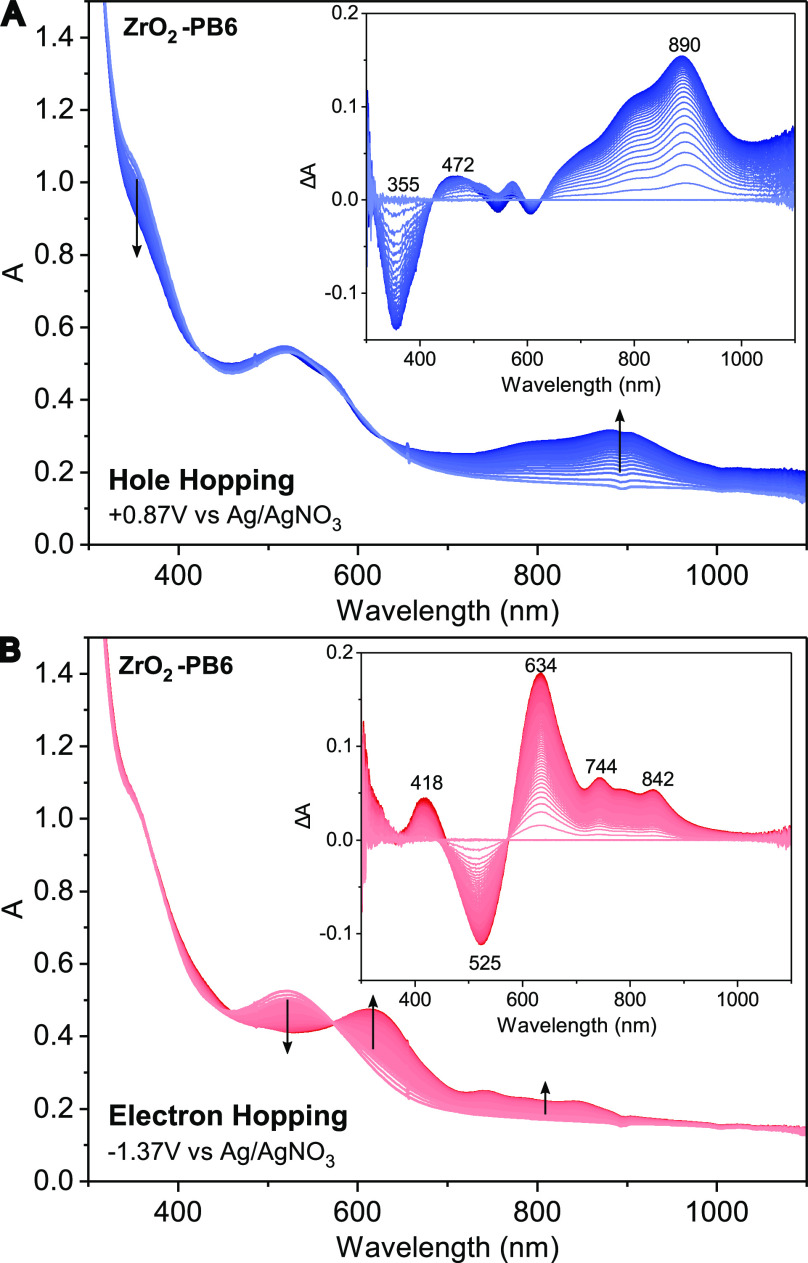
Time-resolved spectroelectrochemistry measurements showing the difference absorption spectra of ZrO_2_-PB6 films in 0.1 M TBAPF_6_ acetonitrile recorded before and after a potential step of (A) +0.87 V and (B) −1.37 V vs Ag/AgNO_3_.

The kinetic traces in acetonitrile (ACN) at 890 nm (PB6^+^) and 634 nm (PB6^•–^) show that hole hopping between PB6 on ZrO_2_ occurs much more rapidly than electron hopping (Figure 4A). In fact, all three solvents and mixtures with ε_*s*_ > 20 in our study (pure ACN, mixture of ACN with dichloromethane (DCM), and mixture of propylene carbonate (PC) with DCM) show faster hole hopping than electron hopping kinetics (Figure 4B). Considering the dye structure and anchoring of PB6 with the TPA subunit closer to the surface, the faster hole hopping is in line with the drop of dielectric constant close to the surface that is reported in the literature. Aramburu-Trošelj et al.^15^ and Bangle et al.^13,14^ showed faster charge hopping kinetics close to the surface in solvents with ε_*s*_ > 25 due to an outer-sphere solvent reorganization energy that was near zero close to the metal oxide surface (∼4 Å) and attributed it to a drastic decrease in solvent dielectric. Further away from the electrode, the reorganization energy increases in agreement with dielectric continuum theory, and at approximately 30 Å away from the surface, the dielectric constant of the respective solvent reaches bulk values.^13−15^ For water, several studies at the liquid–solid interface have also shown that the anomalously low dielectric constant is general to many interfaces and believed to originate from a restricted rotational freedom of the water dipole and a ordered structure of the molecules.^25−28^

**Figure 4:**
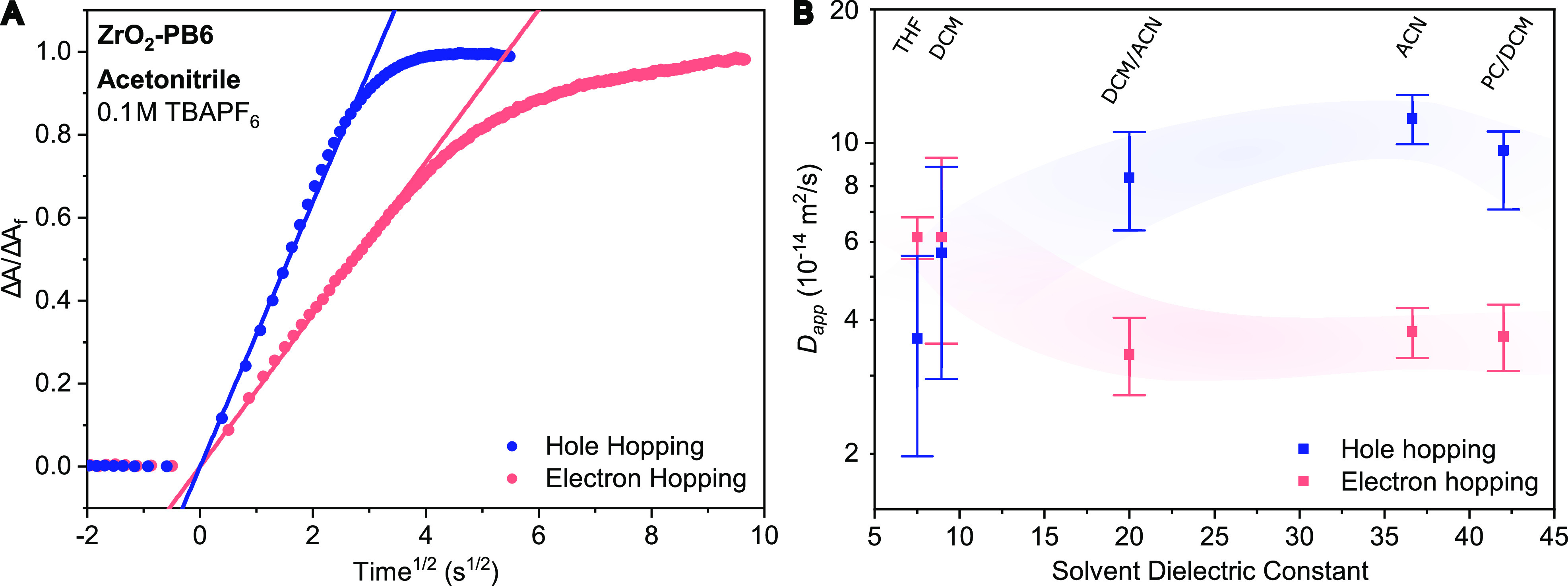
(A) Normalized kinetic traces of ZrO_2_-PB6 in acetonitrile at 890 and 637 nm for hole and electron hopping, respectively. The overlaid linear functions are fits to the Cottrell equation for the initial 60% of total absorbance change. (B) Solvent dependence (expressed through solvent dielectric constant) of the apparent charge diffusion coefficient (*D*_*app*_) extracted from the kinetic traces from electrochemically induced charge hopping of ZrO_2_-PB6 in 0.1 M TBAPF_6_ electrolyte.

With a total size of PB6 of approximately 20 Å, the PMI acceptor should experience only a slightly lower dielectric constant than for bulk ACN according to Bangle et al.^13^ Electron and hole hopping kinetics therefore differ since the PMI acceptor subunit experiences a larger local dielectric constant than TPA, which is in direct contact with the metal oxide surface. Since the inner reorganization energy is expected to be less influenced by solvent changes and is typically much smaller than the outer-sphere solvent reorganization energy,^6^ we can assume that the charge transfer is mainly influenced by the local dielectric constant.^29−32^

The size of the involved subunits can also influence charge transfer kinetics, where a larger involved subunit leads to a lower solvent reorganization energy and thus faster kinetics.^6^ However, considering the somewhat similar size of the two subunits of PB6 (though TPA is slightly smaller), we can exclude that this is the reason for the faster hole hopping rates. Therefore, it can be approximated that the observed difference in hopping rates in ACN, ACN/DCM, and PC/DCM solvent mixtures is mainly due to the different local dielectric constant of high-ε_*s*_ solvents such as ACN and PC in the proximity of the ZrO_2_ surface.

Surprisingly, changing the electrolyte solvent to solvents with a lower ε_*s*_ revealed that the difference in kinetics between hole and electron hopping diminishes (Figure 4B). The similar hole and electron hopping kinetics in tetrahydrofuran (THF) and DCM cannot be explained with the previously discussed model from the literature of solvent behavior at the solid–liquid interface for solvents with ε_*s*_ > 25. Instead, our results indicate that the solvent environment for the TPA donor and PMI acceptor subunit of PB6 is similar in THF and DCM. Interestingly, a threshold dielectric constant at approximately ε_*s*_ ≈ 12 can be extrapolated, below which electron and hole hopping kinetics becomes similar.

Considering the solvent dependence of just the electron hopping in Figure 4B, it can be observed that electron hopping rates increase with a decrease in solvent ε_*s*_. This is because electron hopping between the PMI units occurs further away from the electrode and should experience more bulk-like solvent properties, leading to a lower reorganization energy for solvents with a lower ε_*s*_.^13,14^ However, the solvent dependence of hole hopping across immobilized PB6 on ZrO_2_ is unexpected: it is faster in more polar solvents and slower in solvents with a lower ε_*s*_. This trend is different from what has been observed for solvents with higher ε_*s*_ that have all shown to have a very small solvent barrier close to the metal oxide surface.^13−15^ Since we investigated dry solvents, we can also exclude the possibility of hydrophobic clustering that was observed by Brennan et al.^16^ in aqueous solvents.

The results therefore suggest that solvents with a lower ε_*s*_, such as DCM and THF, do not show the same behavior at the solid–liquid interface as highly polar solvents and that their local dielectric constant remains similar from surface to bulk (Figure 5). Since the drastic decrease in dielectric constant for solvents with high ε_*s*_ is related to a more ordered nature of solvent molecules close to the surface, we assume that solvents with low ε_*s*_ presumably do not form a well-ordered layer at the surface. We therefore hypothesize that solvents below a certain ε_*s*_ threshold do not show large local variations in ε_*s*_ as a function of distance from the surface. Our hypothesis is in line with the work of Daniels et al.,^33^ which shows that the dielectric constant of dimethyl carbonate (ε_*s*_ = 3) exhibits a significantly less drastic decrease of its dielectric constant with increasing electric field strength compared to ACN and PC. While the strength of the electric field may be different, the observed stark effect of PB6 (SI, Figure S10) on ZrO_2_ in the presence a potential that is insufficient to reduce the dye indicates that the dye must lie within the electric double layer, as reported for other photosensitizers.^34^

**Figure 5:**
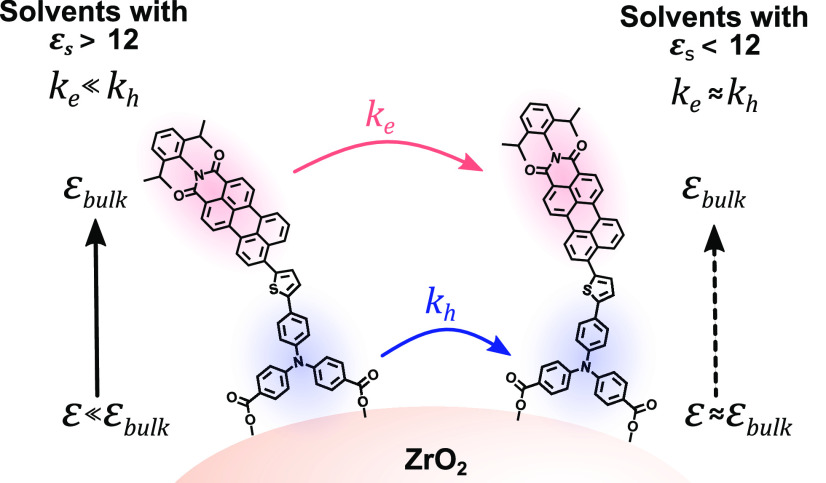
Schematic depiction of the influence of solvent dielectric constant on hole and electron hopping of immobilized PB6 dye on the ZrO_2_ surface.

In conclusion, the charge transport parameters of electron and hole hopping across donor–acceptor organic dyes on an identical mesoporous support in different solvents are reported. We found that the electron hopping mainly happens between the acceptor subunit, and hole hopping between the donor unit of the PB6 dye. While the redox potentials and spectra were mostly unaffected by solvent choice, the hole and electron hopping kinetics were influenced by the solvent. The electron hopping rate, occurring slightly further away from the ZrO_2_ surface, increased when moving to solvents with a lower ε_*s*_, which is expected due to a lower outer-sphere reorganization energy. However, hole hopping did not follow this trend and slowed down when moving toward lower dielectric constant solvents. Since the hole hopping occurs closer to the surface, we hypothesize that the solvent barrier in the electric double layer is only absent in solvents with high dielectric constants and is close to the bulk values for solvents with a low dielectric constant. These factors need to be considered when comparing electron and hole hopping dynamics, as they are greatly influenced by their position within the gradient of environmental changes at the liquid–solid interface. These findings are of interest for advancing our understanding of interfacial charge transport and solvent properties at liquid–solid interfaces in dye-sensitized systems.

## Supporting Information Available

Details of the experimental procedure on the fabrication of sensitized ZrO_2_-PB6 films, BET surface measurements, electrochemistry and spectroelectrochemistry, as well as some theoretical background on charge transfer rates, and additional results, showing the UV–vis absorption of the ZrO_2_-PB6 films, redox potentials, influence of trace oxygen on hopping, dye surface area and loading, calculations of the dielectric constant of solvent mixtures, additional data on electron and hole hopping and Stark effect of ZrO_2_-PB6 films and the charge transfer rates as a function of Pekar factor (PDF)

## ABBREVIATIONS

DSSC: dye-sensitized solar cell
TPA: triphenylamine
PMI: perylene monoimide
DCM: dichloromethane
THF: tetrahydrofuran
FTO: fluorine-doped tin oxide
DPV: differential pulse voltammetry
TBAPF_6_: tetrabutylammonium hexafluorophosphate
ACN: acetonitrile
PC: propylene carbonate
