# Supplementary material for: Lateral Electron and Hole Hopping between Dyes on Mesoporous ZrO2: Unexpected Influence of Solvents with a Low Dielectric Constant
Source: J Am Chem Soc. 2023 May 1;145(21):11472–6. doi: 10.1021/jacs.3c01333 (PMC10236494; doi:10.1021/jacs.3c01333)
Supplement: Supplementary file 1 — ja3c01333_si_001.pdf [file ja3c01333_si_001.pdf]

Supporting Information

for

Lateral Electron and Hole Hopping between Dyes on Mesoporous ZrO_2: Unexpected Influence of Solvents with a Low Dielectric Constant

Sina Wrede[1], Bin Cai[1], Amol Kumar[1], Sascha Ott[1], Haining Tian[1,]*

[1] Department of Chemistry-Ångström Laboratory, Uppsala University, SE-75120 Uppsala, Sweden

Corresponding Author

* E-mail: haining.tian@kemi.uu.se

Table of contents

Experimental section 3

Chemicals 3

Sensitized ZrO_2 Thin Films. 3

Dye loading. 3

BET surface area measurement 3

Electrochemistry 3

Differential pulse voltammetry (DPV) 4

Spectroelectrochemistry 4

Determining formal redox potentials 4

Theory 4

Calculating dielectric constants of binary mixtures with linear model 4

Determining apparent diffusion coefficient D_{app} and charge-transfer rates k_{hop} 5

Results 6

UV-Vis absorption of ZrO_2 and ZrO_2-PB6 films 6

Determining formal redox potentials with DPV and Spectroelectrochemistry 6

Influence of overpotential and trace oxygen on hole and electron hopping kinetics 7

Dye surface area and loading 8

Dielectric constants of DCM/ACN and DCM/PC mixtures 8

Additional data showing the observed trend 9

Comparing spectral response to potential in THF, DCM and ACN 11

Charge self-exchange reaction rates as a function of Pekar factor 13

Approximate distance of PB6 subunits to the surface 13

References 15

Experimental section

Chemicals. All chemicals were provided by Sigma-Aldrich and used directly unless stated otherwise. Hexafluorophosphate (Sigma-Aldrich, electrochemical grade) was recrystallized and dried at 80 °C in vacuum prior to use. ZrO_2 screen printing paste (Solaronix, ZT/SP) was diluted with Terpineol as described below. Dichloromethane and acetonitrile were purified and dried with a solvent purification system and 3Å molecular sieves for 48h. Tetrahydrofuran was used directly from the solvent purification system. Propylene carbonate was dried over 3Å molecular sieves for 48h where the molecular sieves were exchanged twice. The PB6 dye was available from a previous study.[1]

Sensitized ZrO_2 Thin Films. Mesoporous ZrO_2 films were prepared on fluorine-doped tin oxide (FTO)-coated glass substrates (Pilkington TEC15) that was cleaned in successive ultrasonic baths of detergents, deionized water, acetone and ethanol. The mesoporous layer was put directly onto the FTO through screen printing one layer of ZrO_2 paste (ZT/SP, Solaronix) which was diluted with Terpineol (2:1 paste:terpineol). After screen printing, the films were heated at 110 °C for 5 min on the hotplate and annealed directly before the dye bath with the following heating process: 180 °C (10 min), 320 °C (10 min), 390 °C (10 min) and 500 °C (60 min). This resulted in a mesoporous ZrO_2 layer with a thickness of 1.17±0.05 µm. The ZrO_2 films were calcinated the day before measuring and sensitized in a 0.2 mM PB6 dye dichloromethane solution overnight and were rinsed with dichloromethane and dried by N_2 stream before use.

Dye loading. The dye loading of the sensitized films was determined for those from the same batch as those utilized for measurements and were cut to an area of 1.5 cm^2 and de-loaded with a 10 mM phenylphosphonic acid in CH_2Cl_2/MeOH (1:1) overnight in 4 mL solution.[2]

From the dye loading and the BET surface area of the ZrO_2 films, we estimated the average distance δ between the dyes on the surface with **Equation S1**:

$$\delta = \sqrt{\frac{A_{ZrO2,BET} * A_{film} * d_{film}}{n_{Dye} * \mathrm{N_A}}} \quad \text{(Eq. S1)}$$

where $A_{ZrO_2,BET}$ is the surface area of ZrO_2 determined with BET per 1 cm^3 of ZrO_2 film and A_{film} is the area of the electrode mesoporous film, with a thickness of d_{film}, for which de-loading was performed (Glass-FTO-ZrO_2 stack), and n_{Dye} is the number of dyes loaded on the electrode mesoporous film which was determined from the deloading experiment. $\mathrm{N_A}$ is Avogadro's constant.

BET surface area measurement. Surface area measurements of the ZrO_2 mesoporous film were carried out using a Micromeritics ASAP 2060 with N_2 sorption isotherm, recorded at 77 K. Before the measurement, the powder was activated under dynamic vacuum (10^{-4} Pa) using a Micromeritic SmartVacPrep sample preparation unit. The ZrO_2 powder was scraped off ten prepared FTO-ZrO_2 films, with an area of 26.4 cm^2, and collected carefully. The weight for each film was measured and averaged, and compared to the total mass of 10 films.

Electrochemistry. Cyclic voltammetry (CV), differential pulse voltammetry (DPV) and potentiostatic measurements were performed with a three-electrode electrochemical setup in a cuvette, utilizing an AUTOLAB potentiostat (PGSTAT302N) controlled by the software Nova 2.1.4. For the reference electrode, Ag/$AgNO_3$ (1.0 mM $AgNO_3$ acetonitrile solution) was utilized and a platinum wire (Pt) as the counter electrode. FTO-ZrO_2-dye films were used directly as working electrodes, submerged in the electrolyte, 0.1 M Tetrabutylammonium hexafluorophosphate ($TBAPF_6$) in the respective solvent. Before each measurement, the setup was pre-bubbled with solvent saturated argon for >7 min and carried out in argon-saturated electrolyte solution. A fresh sample was used for each measurement in

spectroelectrochemistry. All potentials are given *versus* the used Ag/$AgNO_3$ reference, however, to verify the stability of the system and to enable to conversion to other potentials, ferrocene was used as an internal standard. The potential of ferrocene was stable around 0.12 V vs Ag/$AgNO_3$ in 0.1 M $TBAPF_6$ in acetonitrile.

Differential pulse voltammetry (DPV). A conditioning potential of 0 V was with an equilibration time of 5 s was set before the differential pulse program in the desired potential range with a modulation amplitude of 0.025 V, modulation time of 0.05 s and an interval time of 0.5 s, leading to a scan rate of 10 mV/s.

Spectroelectrochemistry. UV-Vis spectroelectrochemistry measurements were carried out in a home-made electrochemical setup, consisting of a glass cuvette with 1 cm pass-length and stopper designed to hold the three electrodes as described previously, which only has the FTO-ZrO_2-dye films in the optical path. Time resolved spectra (0.5 s time intervals) were recorded with a diode array spectrophotometer (Agilent 8453). Before the constant potential step (chronoamperometry) was applied, the sample was allowed to a pre-equilibrate for 5 s at 0 V. All measurements were carried out by purging with solvent saturated argon for >7 min. New samples as working electrodes were utilized for each measurement and possible dye-de-loading was assessed after each measurement to ensure desorption of the dye did not take place during the measurement. A minimum of 3 measurements (therefore of three different ZrO_2-PB6 films) was conducted for each hole or electron hopping in each solvent and charge hopping rates in acetonitrile was measured each measurement day as a reference. The chosen potential steps for electrochemical induced hole or electron hopping were +0.87 V vs Ag/$AgNO_3$, which is 200 mV more positive than the redox potential PB6/$PB6^+$, and -1.37 V vs Ag/$AgNO_3$, which is 150 mV more negative than the redox potential of PB6/$PB6^-$. A smaller overpotential was chosen for the reduction to avoid any possible degradation of the dye on the film. To ensure the smaller overpotential did not affect the charge transfer rates, we performed a control experiment where the potential step was varied and could not detect a significant change in charge transport rates for different overpotentials (see Figure S4).

Determining formal redox potentials. Formal redox potentials for ITO-$PB6^{0/+}$ and ITO-$PB6^{0/-}$ were determined with the help of spectroelectrochemistry by expressing the spectra at each applied potential as the sum of ITO-$PB6^0$ and ITO-$PB6^+$ spectra (or ITO-$PB6^0$ and ITO-$PB6^-$). Spectral features were chosen that are not present in ITO-$PB6^0$, which made it possible to extract the mole fraction of each species χ. The dependence of χ versus the applied potential E_{app} was plotted and fitted with **Equation S2** where E_f is the formal redox potential and α is a non-ideality factor that is typically used for mesoporous films.[3]

$$\chi = \frac{1}{1+10^{(E_{app}-E_f)/(59.2\alpha)}} \quad \text{(Eq. S2)}$$

Theory

Calculating dielectric constants of binary mixtures with linear model. The dielectric constant of solvent mixtures ε_m can be approximated with a linear combination of the pure solvents ε_i, the molar volumes υ_i and the molar fractions χ_i, as given in **Equation S3**:[4]

$$\varepsilon_m = \frac{\sum_i^n \varepsilon_i \chi_i \upsilon_i}{\sum_i^n \chi_i \upsilon_i} \quad \text{(Eq. S3)}$$

The molar volume can be calculated from the pure solvent molar masses M_i, and their densities ρ_i.[5]

$$\upsilon_i = \frac{M_i}{\rho_i} \quad \text{(Eq. S4)}$$

Since we mixed two volumes, instead of plotting the dielectric constant of the mixture ε_m against the molar fraction χ_i, we have expressed the function as a plot against volume fraction v_i:

$$\chi_i = \frac{n_i}{\sum_i^n n_i} = \frac{\frac{\mathrm{v}_i}{v_i}}{\sum_i^n \frac{\mathrm{v}_i}{v_i}} \quad \text{(Eq. S5)}$$

Determining apparent diffusion coefficient *D_{app}* and charge-transfer rates *k_{hop}*. A modified version of the Cottrell equation is commonly used to relate the observed change in absorption during Spectroelectrochemistry with the apparent diffusion coefficient throughout the film, as given in **Equation S6**:[6]

$$\frac{\Delta A}{\Delta A_f} = \frac{2\sqrt{t\, D_{app}}}{d\sqrt{\pi}} \quad \text{(Eq. S6)}$$

where ΔA is the observed change in absorption after the potential step with time t, ΔA_f is the final or rather maximal possible absorbance change (the equation is only valid in the limit of $\Delta A \leq \Delta A_f$) and D_{app} is the apparent diffusion coefficient throughout the film with a thickness d.

From the experimental data, the apparent diffusion coefficient in the film, D_{app}, can be extracted from the slope of the initial 60% of a plot of $\frac{\Delta A}{\Delta A_f}$ versus the square root of time. Only the first 60% of total absorption change are fitted since the photosensitizer is bound to the metal oxide surface and cannot diffuse away from the surface – in contrast to the classical interpretation of the Cottrell equation that uses a semi-infinite diffusion boundary. Despite this, it has been shown that the linear behavior is maintained for sensitized films for first 60%, after which the data deviates from the predicted linear relationship (once the frontline of the reduced or oxidized molecules reaches the outer edge of the film).[6–8]

If we consider a uniform arrangement of the sensitizer on the surface, the apparent diffusion coefficient D_{app} through the film can then be used to calculate the intermolecular hopping rate k_{hop} between molecules on the surface with **Equation S7**:[9]

$$D_{app} = \frac{k_{hop}\delta^2}{2n} \quad \text{(Eq. S7)}$$

where δ is the intermolecular distance and n the dimensionality of the hopping process, which we assume to be 3-dimensional, so n=3. It is likely that in reality n lies somewhere between 2 and 3 as we have hopping across a surface (2D) of a 3-dimensional film with defects, tortuosity of the surfaces, constricted interparticle connections and incomplete connectivity between dyes. However, this does not change the calculated hopping rate too significantly and has been shown to lie within the error of the measurement for a sensitized NiO surface.[10]

Results

UV-Vis absorption of ZrO_2 and ZrO_2-PB6 films

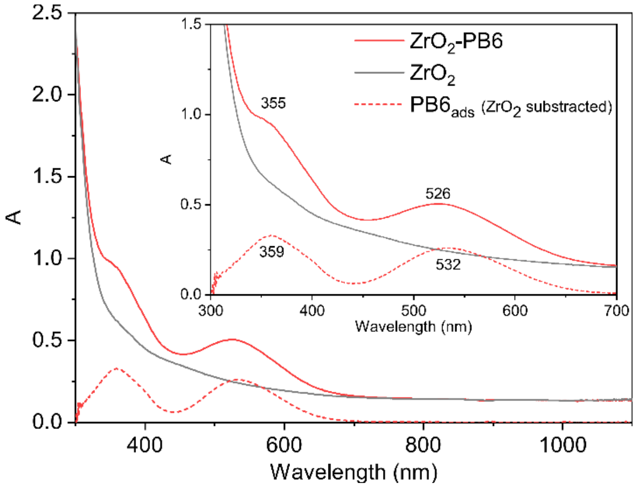

Figure S1. UV-Vis steady-state absorption of ZrO_2-PB6 films submerged in 0.1M $TBAPF_6$ in dichloromethane, as well as $PB6_{ads}$ which was obtained by subtracting the spectrum of ZrO_2 from ZrO_2-PB6.

Determining formal redox potentials with DPV and Spectroelectrochemistry

The redox potentials for $PB6^{0/+}$ and $PB6^{0/-}$ on a mesoporous support were determined with the help of DPV of ZrO_2-PB6 films in 0.1 M $TBAPF_6$ in acetonitrile (ACN). While the peak of $PB6^{0/-}$ on ZrO_2 in ACN is relatively narrow, the peak of $PB6^{0/+}$ is broader (**Figure S2A**). This is due to the merging of first and second oxidation peak in ACN on ZrO_2, as it is known from previous studies that PB6 has a second oxidation peak close to the first.[1] To determine the redox potential of $PB6^{0/+}$ on film, spectroelectrochemical measurements on ITO were employed (**Figure S3**). The mole fraction χ versus the applied potential E_{app} was fit to Eq. S1 and gave a formal redox potential of 0.67 V vs $Ag/AgNO_3$ for the first oxidation, $PB6^{0/+}$, where the onset of spectral features of the first (900 nm peak) and second oxidation (broad at 1050 nm) could be clearly distinguished. The formal redox potential for $PB6^{0/-}$ on mesoporous ITO from the fit, -1.22 V, matched the value obtained by DPV on ZrO_2 support. DPV of

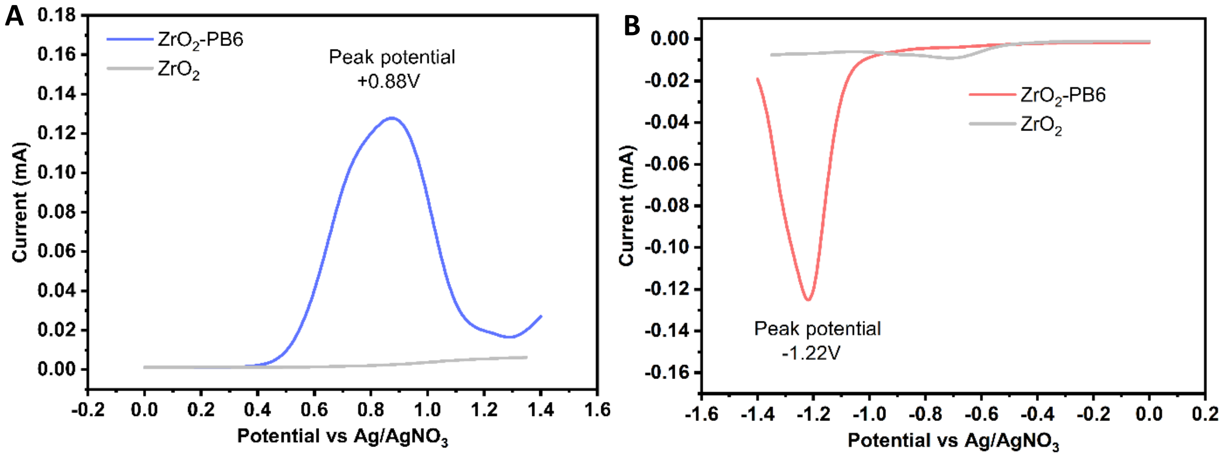

Figure S2. DPV of ZrO_2-PB6 films in 0.1 M $TBAPF_6$ in acetonitrile with a scan rate of 10 mV/s in the (A) positive and (B) negative potential range.

PB6 in tetrahydrofuran (THF) and dichloromethane (DCM) were also measured and showed no significant deviation of peak potentials.

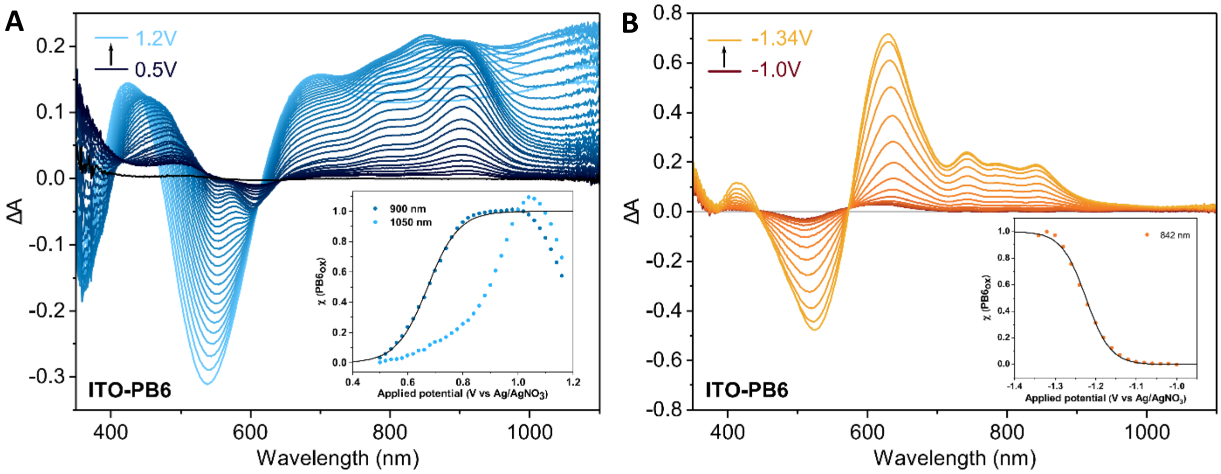

Figure S3. Spectroelectrochemical determination of formal redox potentials, showing the UV-Vis absorption spectra of ITO-PB6 films in 0.1 M $TBAPF_6$ in ACN upon the application of the indicated potential range with a step of 20 mV. The insets show the mole fraction of (A) $PB6^{+}$ or (B) $PB6^{\cdot -}$ as a function of the applied potential with an overlaid fit to Eq. S2.

Influence of overpotential and trace oxygen on hole and electron hopping kinetics

To ensure the smaller overpotential for the electron hopping, to avoid possible dye degradation, did not affect the charge transfer rates, we performed a control experiment where the potential step was varied and could not detect a significant change in charge transport rates with varying overpotentials (**Figure S4**)

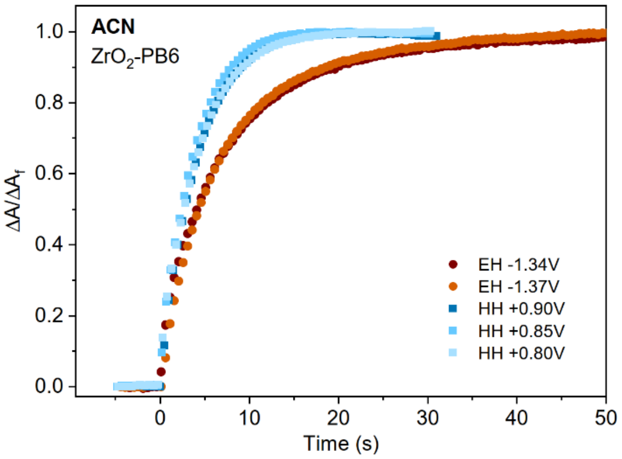

Figure S4: Normalized kinetic traces of ZrO_2-PB6 films in 0.1M $TBAPF_6$ acetonitrile with different overpotentials at 890 nm and 637 nm for hole and electron hopping, respectively.

To ensure our hopping kinetics were not strongly affected by trace oxygen (especially for electron hopping), we compared the hopping kinetics inside the glovebox and outside the glovebox where the setup was purged with argon (**Figure S5**). If trace oxygen would influence our kinetics, hopping rates in the glovebox would be expected to be faster, which could not be confirmed.

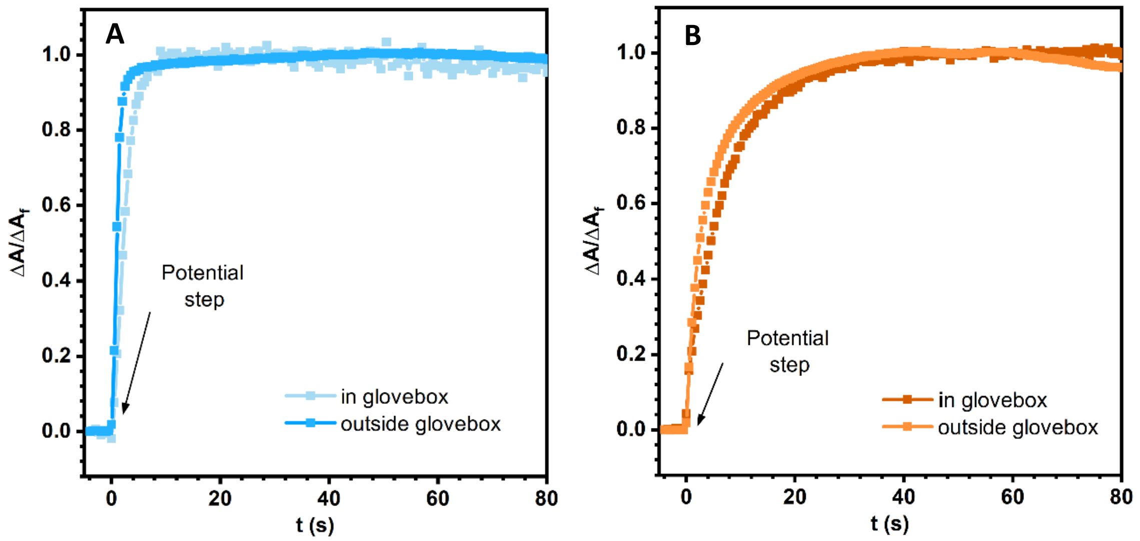

Figure S5. Kinetic traces of the emergence of (A) $PB6^{+}$ and (B) $PB6^{\bullet-}$ after a potential step of (A) +0.87 V and (B) -1.37 V vs $Ag/AgNO_3$, both measured in the glovebox and outside the glovebox where the electrochemical setup was purged with argon. These films were measured of a different ZrO_2-PB6 batch than in the main text and therefore have slightly different loading and thickness, which influences hopping kinetics.

Dye surface area and loading

The absorption spectra of the phenylphosphonic acid solutions from de-loaded ZrO_2-PB6 films were recorded and used to calculate the quantity of dye in solution, and thus on film, which was $6.9 \cdot 10^{-5}$ mol/cm^3 with a film thickness of 1.17 µm.

From BET measurements of ZrO_2 powder from the films, a surface area of 43.1 m^{2}g^{-1} was determined. Together with the weight (4.7±0.2 mg) and area (26.4 cm^2) of a single scraped off films, this resulted in a surface area per electrode film area of 76±3 cm^2/cm^2 and per mesoporous ZrO_2 volume of approximately 65 m^2/cm^3. With the surface area of the ZrO_2 film and the dye loading and equation S1, it was possible to calculate the intermolecular distance of the PB6 dye molecules, approximately 12 Å.

Dielectric constants of DCM/ACN and DCM/PC mixtures

With a simple linear model using **Eq S3-S5**, for the binary mixtures (**Figure S6)**, we can extract that a volumetric 1:1 mixture of DCM/ACN yields a dielectric constant of 20, and a DCM/PC mixture with approximately 45% v/v PC gives a dielectric constant of 42. The dielectric constants used for the solvent dependent hopping are summarized in **Table S1**.

Table S1: Dielectric constants of the pure solvents and binary mixtures that were used in this study.

Solvent	Dielectric constant ε
Acetonitrile	36.6[a]
Dichloromethane	8.9[a]
Tetrahydrofuran	7.62[b]
Propylene Carbonate	64.9[c]
PC/DCM (45 %v/v)	42
ACN/DCM (50 %v/v)	20

[a] From reference [14]. [b] From reference [15]. [c] From reference [16].

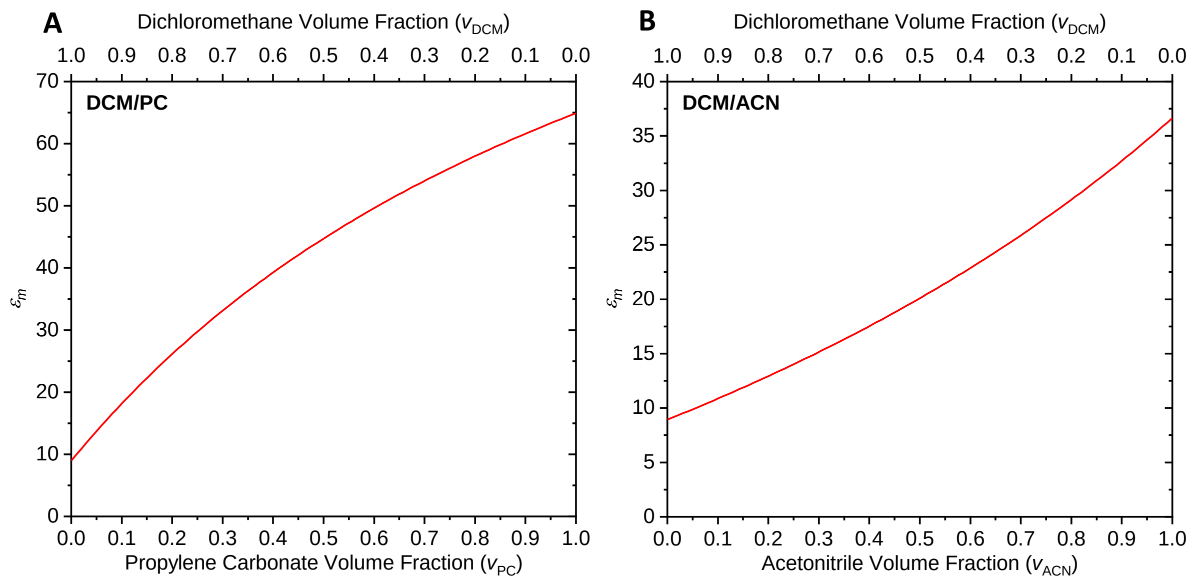

Figure S6. Dielectric constant of the binary mixtures (A) DCM/PC and (B) DCM/ACN depending on their volume fraction, calculated with the linear model equation S2.

Charge transfer parameters for ZrO_2-PB6 films in ACN and THF

The extracted apparent charge diffusion coefficients D_{app} that are depicted in Figure 3D (main text) for hole and electron hopping from the linear fit are summarized in **Table S2** for ACN and THF, as well as the intermolecular charge hopping rates k_{hop} calculated with Eq. S7.

Table S2. Charge transfer parameters for ZrO_2-PB6 films in ACN and THF. The apparent diffusion rate (D_{app}) was determined from the kinetic traces and the intermolecular hopping rates k_{hop} for three-dimensional hopping across the ZrO_2 surface was calculated with equation S7 with an intermolecular distance δ of 12 Å.

	Dielectric constant	Electron hopping D_{app} (m^2s^{-1})	Electron hopping k_{hop} (s^{-1})	Hole hopping D_{app} (m^2s^{-1})	Hole hopping k_{hop} (s^{-1})	Error
ACN	36.6[a]	$3.76\cdot10^{-14}$	$1.6\cdot10^{5}$	$1.13\cdot10^{-13}$	$4.7\cdot10^{5}$	10 %
THF	7.6[b]	$6.14\cdot10^{-14}$	$2.6\cdot10^{5}$	$3.85\cdot10^{-14}$	$1.5\cdot10^{5}$	~15%

[a] From reference [14]. [b] From reference [15].

Additional data showing the observed trend

To ensure maximum comparability between ZrO_2-PB6 films, the data for the figure in the main text (Figure 4A) is from 5 ZrO_2-films (each yielding 10 ZrO_2-PB6 films) that were fabricated on the same day and then calcinated and sensitized in the exact same staining solution the day before measuring. Therefore, additional data that is from ZrO_2 films fabricated on a different day and sensitization solution were not included in the figure in the main text since they showed a slightly different thickness and dye loading which affects hopping rates. However, they still show the same trend and show that even despite the large error in Figure 4A in the main text, the unexpected solvent behavior of low dielectric solvents is reproducible and real. The additional data shows the same observed trend that hole hopping in DCM is slower than in ACN (**Figure S7**) and that electron hopping in ACN was slower than in THF (**Figure S8**).

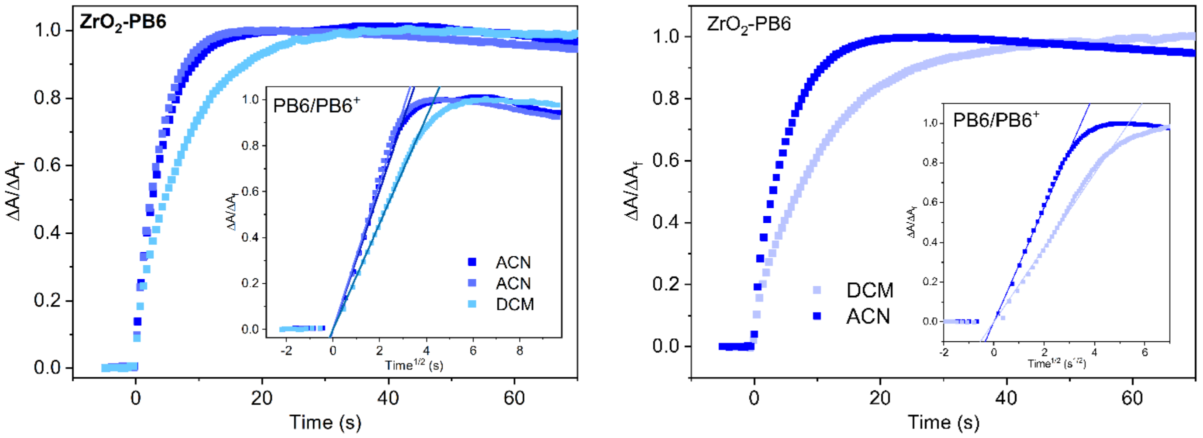

Figure S7. Normalized kinetic traces of the emergence of $PB6^+$ after a potential step of +0.87 V vs $Ag/AgNO_3$, comparing the hole hopping rate in ACN and DCM of two different batches. These films were measured of a different ZrO_2-PB6 batch than in the main text and therefore have slightly different loading and thickness, which influences hopping kinetics, however the observed trend is the same.

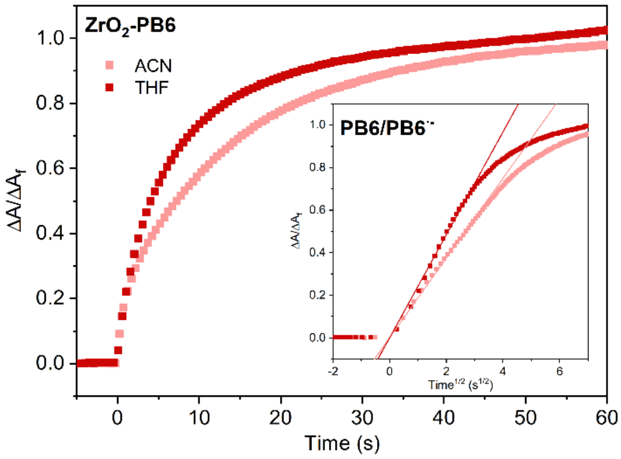

Figure S8. Normalized kinetic traces of the emergence of $PB6^{\bullet-}$ after a potential step of -1.37 V vs $Ag/AgNO_3$, comparing the electron hopping rate in ACN and THF. These films were measured of a different ZrO_2-PB6 batch than in the main text and therefore have slightly different loading and thickness, which influences the actual rates for hopping kinetics, however the observed trend is the same.

Comparing spectral response to potential in THF, DCM and ACN

A factor that can influence hopping rates is solvation of the dyes in solution, which is affecting the electronic structure and thus reflects the reorganization of the dyes. From recorded spectra of ZrO_2-PB6 in THF, DCM and ACN (see **Figure S9**) we could observe a very slightly higher absorption of PB6 in its ground state on ZrO_2 when moving towards lower dielectric constants, which could indicate a better solvation of PB6 in THF and DCM compared to ACN. However, since the donor and acceptor should be somewhat similarly affected, this does not fully explain the opposite solvent dependence we observe for hole and electron hopping of PB6 on ZrO_2.

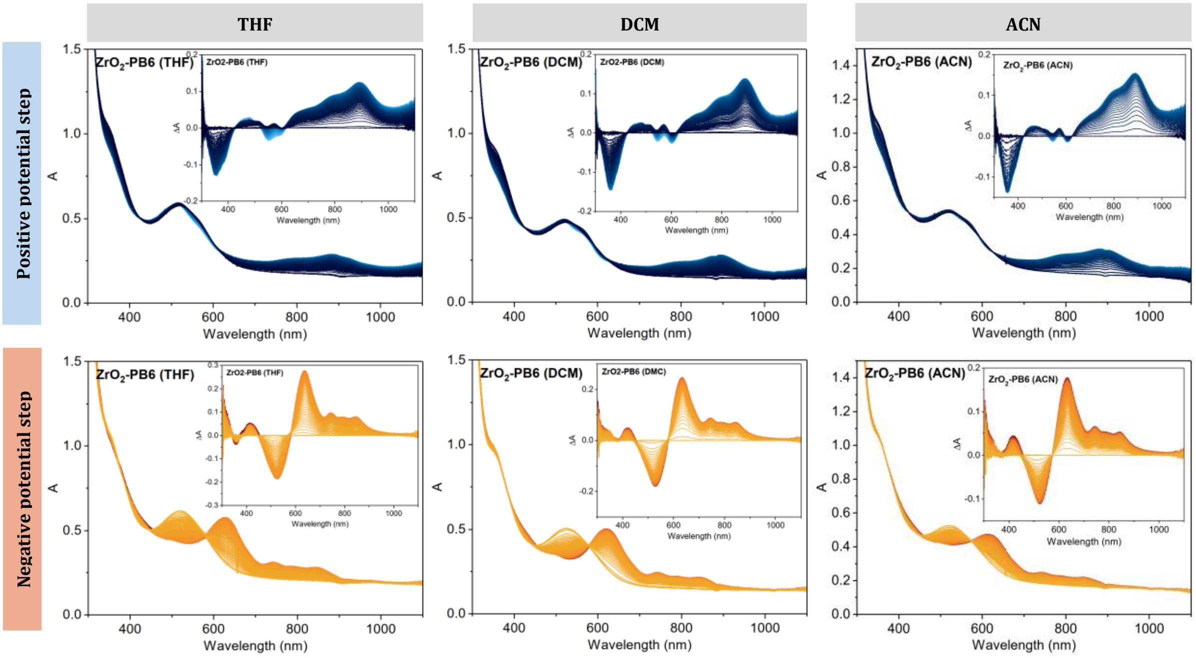

Figure S9. Spectral response to (top row) positive applied potential +0.87 V vs Ag/$AgNO_3$ or (bottom row) negative potential step (-1.37 V vs Ag/$AgNO_3$) of ZrO_2-PB6 films in 0.1M $TBAPF_6$ in THF, DCM and ACN.

Stark effect of ZrO_2-PB6

To confirm if the PB6 dye lies within the Helmholtz layer, we investigated the Stark effect of ZrO_2-PB6 films. Recorded spectra of the ZrO_2-PB6 films in $TBAPF_6$ in ACN with an applied potential that is not sufficient to reduce the dye show a characteristic bathochromic Stark shift (**Figure S10**). The shape of the first derivative of the PB6 absorption spectrum shows that the effect is a first order Stark-Effect where the external field $\Delta\vec{E}$ and the dipole of the dye $\Delta\vec{\mu}$ have the same sign, since the observed Stark Effect follows the negative first derivative of the absorption, $\Delta A \sim -dA/d\lambda$.

From the observed amplitude and direction of the Stark-Shift, we believe that the majority of the dyes are experiencing the same field, that is perpendicular to the ZrO_2 surface and not only the ones close the FTO interface. To confirm that the majority of the dyes on the ZrO_2 are contributing to the Stark Shift and not only the ones close to the FTO in **Figure S10**, we have performed an additional measurement of PB6 sensitized FTO films (FTO-PB6) where the potential was varied (**Figure S11**). To verify that there is some PB6 on the FTO surface, we have also applied a potential that is sufficient to reduce the PB6 (-1.3V). The amplitude of the signal at -0.9V that looks like Stark-Shift of FTO-PB6 films is less than 1% of that observed for ZrO_2-PB6 films which means that the dyes absorbed on ZrO_2 are the ones giving the signal for the Stark shift in ZrO_2-PB6 films. Potential induced Stark-Shift (or

electroabsorption) of dyes on TiO_2 is typically attributed to the accumulation of electrons in the conduction band or in trapped states.[11] It is possible that the observed Stark-Shift of dye sensitized ZrO_2 is of similar origin since ZrO_2 nanoparticles are known to exhibit trapped states on their surface.[12] From the data, we therefore conclude that the observed Stark-Shift is from the dye on the ZrO_2 surface and the main take-away from the observed Stark Shift is that the PB6 dye lies within the electric double layer.

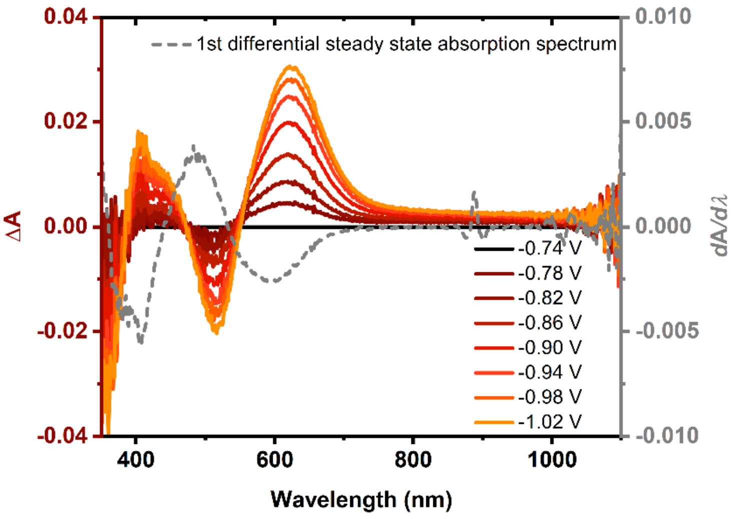

Figure S10: Spectral response of ZrO_2-PB6 films to a negative applied potential in V vs $Ag/AgNO_3$ that is not sufficient to reduce the dye in 0.1M $TBAPF_6$ in ACN, together with the first derivative of the steady state absorption spectrum of ZrO_2 surface adsorbed PB6.

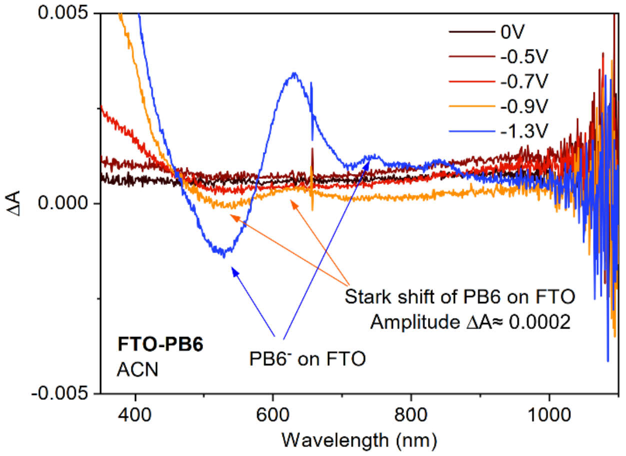

Figure S11: Spectral response of FTO-PB6 film, in 0.1M $TBAPF_6$ in ACN, to a negative applied potential in V vs $Ag/AgNO_3$.

Charge self-exchange reaction rates as a function of Pekar factor

Charge self-exchange reaction rates are often plotted against the Pekar factor $\gamma = \left(\frac{1}{n^2} - \frac{1}{\varepsilon}\right)$ where n is the refractive index, and ε the static dielectric constant of the solvent (bulk value). Since it has been recognized that the solvent relaxation time τ_L influences the rate constant,[13] the solvent Pekar factor is plotted against $\log\left(k\tau_L\gamma^{-½}\right)$ where k is the measured rate constant in **Figure S12**. Since τ_L is unknown for PC and its solvent mixtures, only THF, DCM and ACN were plotted, where τ_L was taken from Ref. 13. For the DCM/ACN mixture, τ_L was taken as the average of the two solvent relaxation times.

As expected, the electron self-exchange reaction of PB6/PB6$^-$ follows a linear solvent dependence since it is further away from the ZrO_2 surface and experiences bulk-like solvent properties. While the electronic coupling and molecular radius is expected to differ slightly between the donor and acceptor subunit that are involved in hole or electron hopping, the linear regression of the electron self-exchange reaction of PB6/PB6$^-$ was used to at least roughly estimate the dielectric constant that the donor subunit of PB6 experiences during hole hopping. With these assumptions, the dielectric constant of THF and DCM were similar to their bulk values while the dielectric constant for acetonitrile needs to be significantly lower, approximately $\epsilon \approx \frac{1}{4}\epsilon_{bulk}$ to reach the measured rate constant. Though this is a rough estimation, it does support the hypothesis that the dielectric constant for low dielectric solvents such as THF and DCM does not significantly change from bulk to surface in contrast to large dielectric constant solvents.

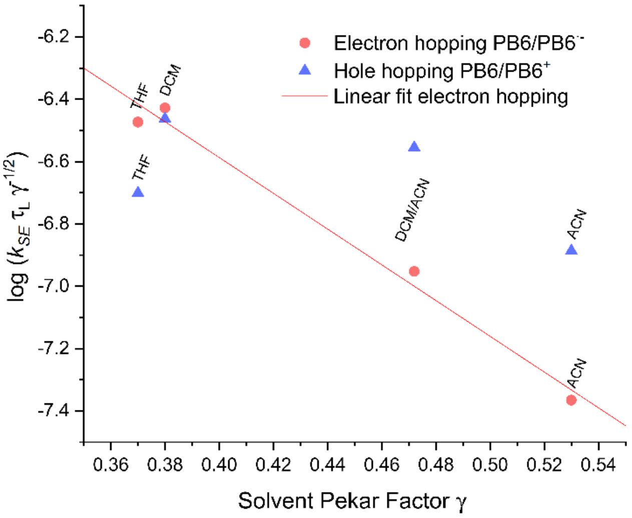

Figure S42. Solvent dependence of the electron self-exchange reaction of PB6/PB6$^-$ (red) and PB6/PB6$^+$ (blue).

Approximate distance of PB6 subunits to the surface

The relative positions to the ZrO_2 surface were roughly approximated from a 3D model of PB6. The approximate distance from the surface to the center of the TPA subunit (colored in blue) is 3.5 Å and 18 Å to the center of the PMI subunit (colored in red).

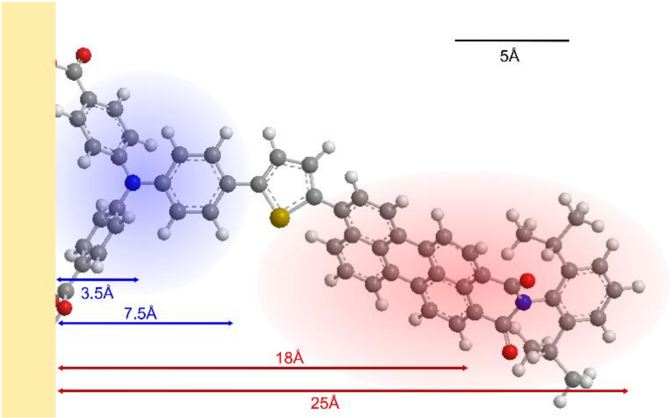

Figure 13: 3D model of PB6 that was used to approximate the distances of the TPA (blue) and PMI (red) subunit to the ZrO_2 surface.

References

(1) Tian, L.; Föhlinger, J.; Pati, P. B.; Zhang, Z.; Lin, J.; Yang, W.; Johansson, M.; Kubart, T.; Sun, J.; Boschloo, G.; Hammarström, L.; Tian, H. SI: Ultrafast Dye Regeneration in a Core–Shell NiO–Dye–TiO_2 Mesoporous Film. *Phys. Chem. Chem. Phys.* **2018**, *20* (46), 29566–29566, DOI:10.1039/c8cp91912g.

(2) Ameline, D.; Diring, S.; Farre, Y.; Pellegrin, Y.; Naponiello, G.; Blart, E.; Charrier, B.; Dini, D.; Jacquemin, D.; Odobel, F. Isoindigo Derivatives for Application in P-Type Dye Sensitized Solar Cells. *RSC Adv.* **2015**, *5* (104), 85530–85539, DOI:10.1039/c5ra11744e.

(3) Bangle, R. E.; Schneider, J.; Conroy, D. T.; Aramburu-Trošelj, B. M.; Meyer, G. J. Kinetic Evidence That the Solvent Barrier for Electron Transfer Is Absent in the Electric Double Layer. *J. Am. Chem. Soc.* **2020**, *142* (35), 14940–14946, DOI:10.1021/jacs.0c05226.

(4) Hall, D. S.; Self, J.; Dahn, J. R. Dielectric Constants for Quantum Chemistry and Li-Ion Batteries: Solvent Blends of Ethylene Carbonate and Ethyl Methyl Carbonate. *J. Phys. Chem. C* **2015**, *119* (39), 22322–22330, DOI:10.1021/acs.jpcc.5b06022.

(5) Ding, M. S.; Xu, K.; Jow, T. R. Liquid-Solid Phase Diagrams of Binary Carbonates for Lithium Batteries. *J. Electrochem. Soc.* **2000**, *147* (5), 1688, DOI:10.1149/1.1393419.

(6) Bonhôte, P.; Gogniat, E.; Tingry, S.; Barbé, C.; Vlachopoulos, N.; Lenzmann, F.; Comte, P.; Grätzel, M. Efficient Lateral Electron Transport inside a Monolayer of Aromatic Amines Anchored on Nanocrystalline Metal Oxide Films. *J. Phys. Chem. B* **1998**, *102* (9), 1498–1507, DOI:10.1021/jp972890j.

(7) Trammell, S. A.; Meyer, T. J. Diffusional Mediation of Surface Electron Transfer on TiO_2. *J. Phys. Chem. B* **1999**, *103* (1), 104–107, DOI:10.1021/jp9825258.

(8) DiMarco, B. N.; Motley, T. C.; Balok, R. S.; Li, G.; Siegler, M. A.; O'Donnell, R. M.; Hu, K.; Meyer, G. J. A Distance Dependence to Lateral Self-Exchange across Nanocrystalline TiO_2. A Comparative Study of Three Homologous Ru III/II Polypyridyl Compounds. *J. Phys. Chem. C* **2016**, *120* (26), 14226–14235, DOI:10.1021/acs.jpcc.6b04438.

(9) Ruff, I.; Botár, L. Effect of Exchange Reactions on Transport Processes: A Comparison of Thermodynamic Treatment with Random Walk on Lattice Points. *J. Chem. Phys.* **1985**, *83* (3), 1292–1297, DOI:10.1063/1.449445.

(10) Wrede, S.; He, L.; Boschloo, G.; Hammarström, L.; Kloo, L.; Tian, H. Electron-Hopping across Dye-Sensitized Mesoporous NiO Surfaces. *Phys. Chem. Chem. Phys.* **2022**, *24* (48), 29850–29861, DOI:10.1039/D2CP03249J.

(11) Yang, W.; Hao, Y.; Vlachopoulos, N.; Eriksson, A. I. K.; Boschloo, G. Studies on the Interfacial Electric Field and Stark Effect at the TiO_2/Dye/Electrolyte Interface. *J. Phys. Chem. C* **2016**, *120* (39), 22215–22224, DOI:10.1021/acs.jpcc.6b07096.

(12) Rath, M. C.; Ramakrishna, G.; Mukherjee, T.; Ghosh, H. N. Electron Injection into the Surface States of ZrO_2 Nanoparticles from Photoexcited Quinizarin and Its Derivatives: Effect of Surface Modification. *J. Phys. Chem. B* **2005**, *109* (43), 20485–20492, DOI:10.1021/jp0533980.

(13) Grampp, G.; Harrer, W.; Jaenicke, W. The Role of Solvent Reorganization Dynamics in Homogeneous Electron Self-Exchange Reactions. *J. Chem. Soc. Faraday Trans. 1 Phys. Chem. Condens. Phases* **1987**, *83* (1), 161–166, DOI:10.1039/F19878300161.

(14) Lide, D. R. *CRC Handbook of Chemistry and Physics, 85th Edition*; 2004; Vol. 85.

(15) Gill, D. S.; Singh, J.; Ludwig, R.; Zeidler, M. D. Nuclear Magnetic Resonance Relaxation, Permittivity, Viscosity and Ultrasonic Velocity Measurements in Binary Mixtures of Methanol

and Tetrahydrofuran. *J. Chem. Soc. Faraday Trans.* **1993**, *89* (21), 3955–3958, DOI:10.1039/FT9938903955.

(16) Côté, J.-F.; Brouillette, D.; Desnoyers, J. E.; Rouleau, J.-F.; St-Arnaud, J.-M.; Perron, G. Dielectric Constants of Acetonitrile, γ-Butyrolactone, Propylene Carbonate, and 1,2-Dimethoxyethane as a Function of Pressure and Temperature. *J. Solution Chem.* **1996**, *25* (12), 1163–1173, DOI:10.1007/BF00972644.
